# Supplementary figures and images for: Postinhibitory excitation in motoneurons can be facilitated by hyperpolarization-activated inward currents: A simulation study
Source: PLoS Comput Biol. 2024 Jan 19;20(1):e1011487. doi: 10.1371/journal.pcbi.1011487 (PMC10843122; doi:10.1371/journal.pcbi.1011487)

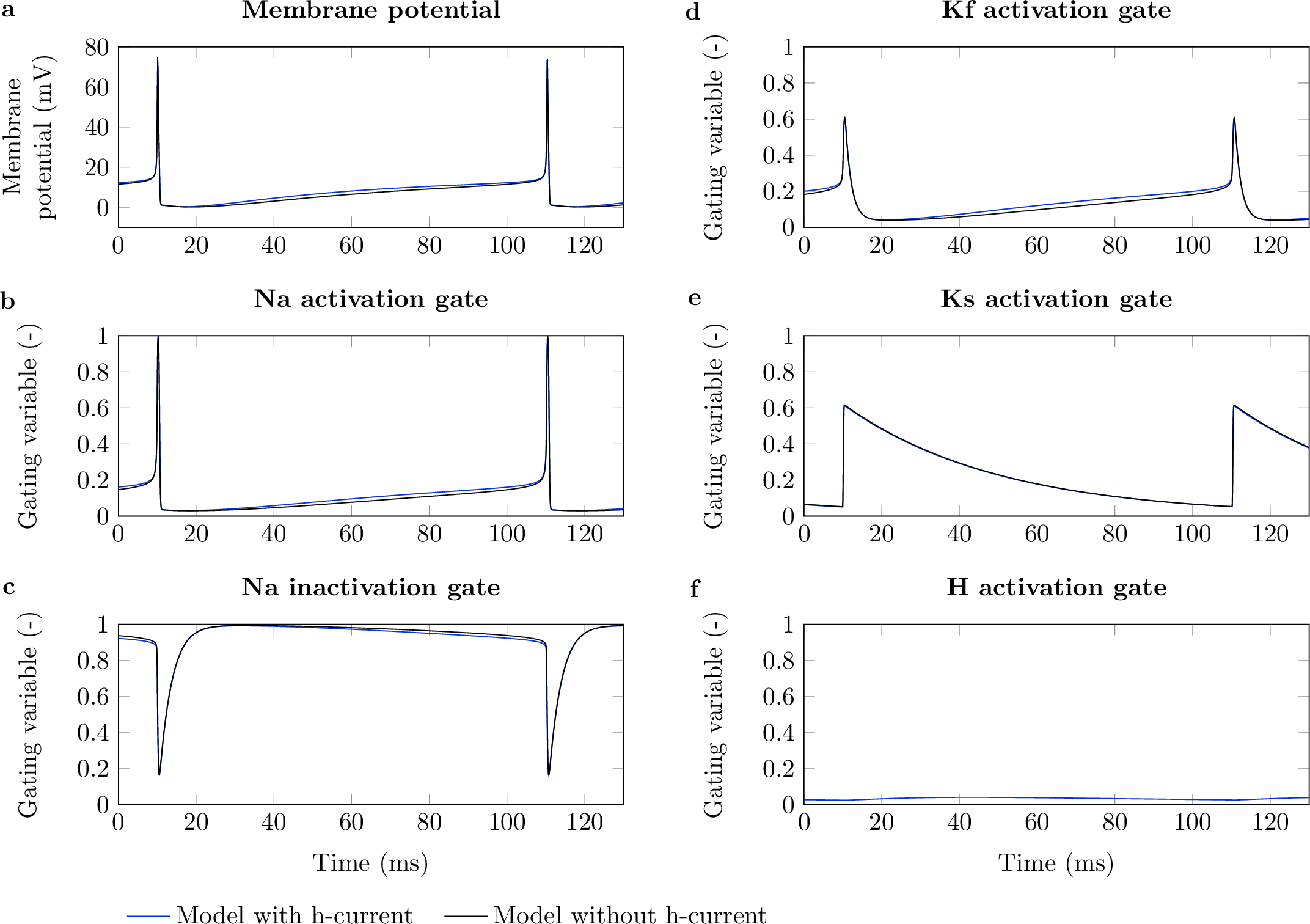

Supplement: S1 Fig — Motoneuron model gating variables for one interspike interval (duration 90.3 ms) of the model with (blue) and without (black) h-current. Shown are membrane potential (a), sodium channel (Na) activation gate (b), Na inactivation gate (c), fast potassium channels (Kf) activation gate (d), slow potassium channel (Ks) activation gate (e) and h-channel (H) activation gate (f). The gating variables are defined as described in [18] and [38]. (TIF) [file pcbi.1011487.s001.tif]

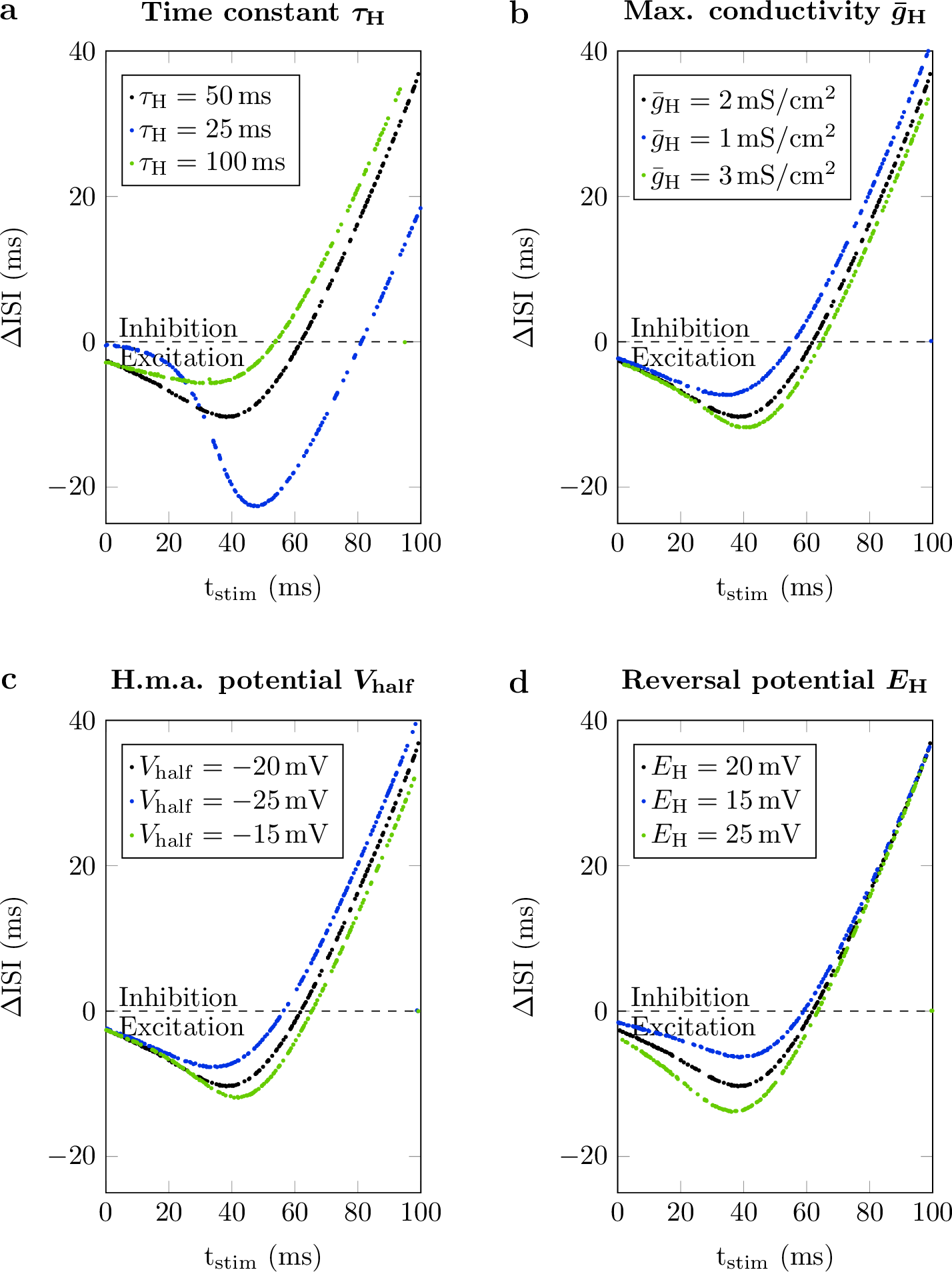

Supplement: S2 Fig — Change of interspike interval duration (Δ ISI) over time of stimulus (IPSP) application with respect to the last discharge (tstim). Default parameters are shown in black. In the simulations all parameters were fixed except for time constant τH (a), maximum conductance of h-current g¯H (b), half-maximum activation potential Vhalf (c) or reversal potential EH (d). Baseline frequency 10 Hz, no noise, inhibitory stimulus amplitude -10 nA. (TIF) [file pcbi.1011487.s002.tif]

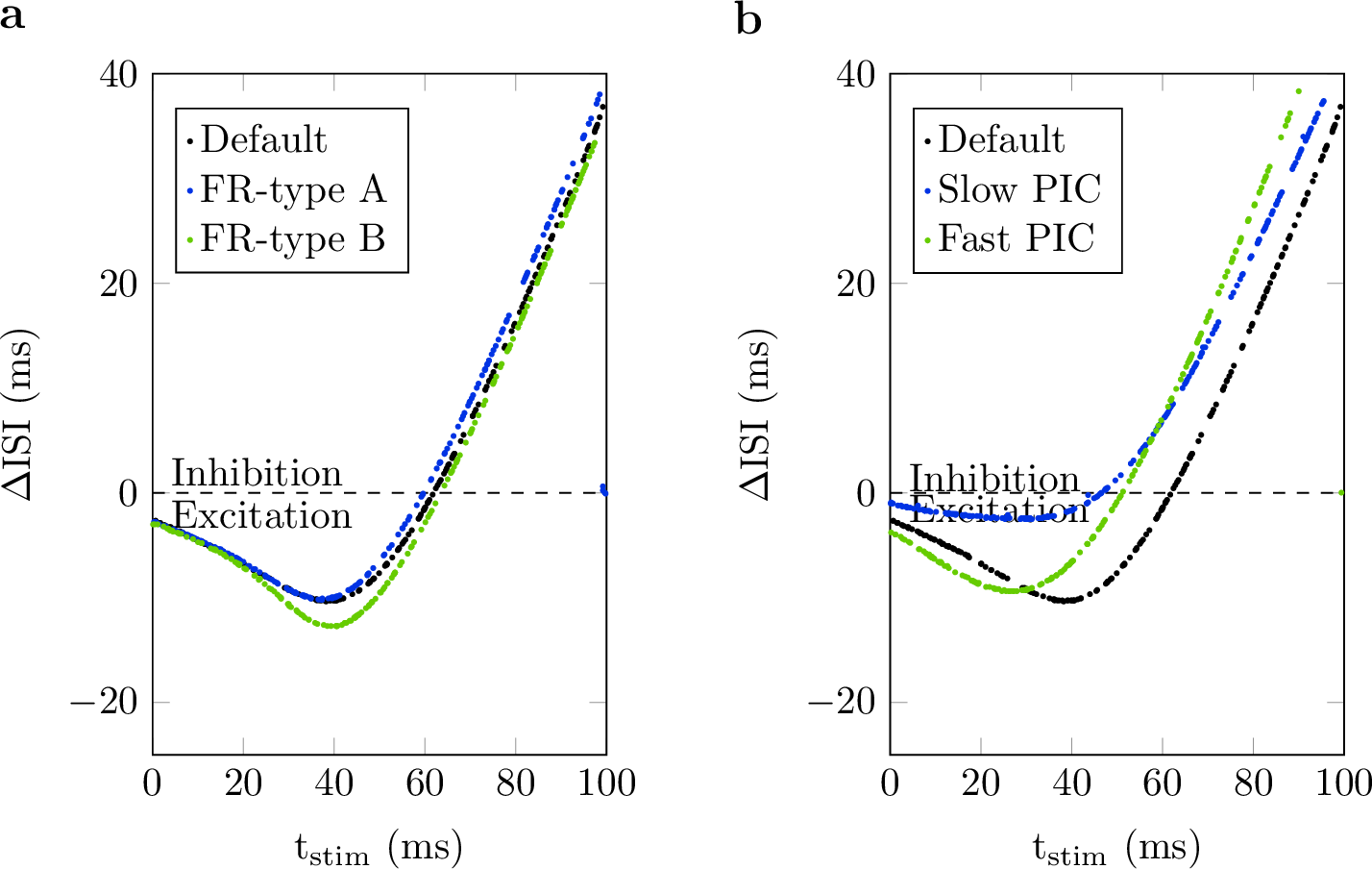

Supplement: S3 Fig — Change of interspike interval duration (Δ ISI) over time of stimulus (IPSP) application with respect to the last discharge (tstim). Default parameters are shown in black. (a): Variation of the motoneuron size. FR-type motoneurons correspond to motoneurons with size-dependent parameters according to the mean values for FR-type neurons in [17]. Version A and B employ a maximum h-channel conductance of g¯H,A=2mscm-2 and g¯H,B=4mscm-2, respectively. (b): Influence of persistent inward currents (PICs) injected into the dendrite compartment of the motoneuron model. PIC dynamics according to [24]. Maximum PIC conductance 0.1 mScm-2. Slow PIC: time constant 100 ms, reversal potential 140 mV. Fast PIC: time constant 1 ms, reversal potential 120 mV. Baseline frequency 10 Hz, no noise, inhibitory postsynaptic current amplitude -10 nA. (TIF) [file pcbi.1011487.s003.tif]
