# Supplementary material for: Postinhibitory excitation in motoneurons can be facilitated by hyperpolarization-activated inward currents: A simulation study
Source: PLoS Comput Biol. 2024 Jan 19;20(1):e1011487. doi: 10.1371/journal.pcbi.1011487 (PMC10843122; doi:10.1371/journal.pcbi.1011487)
Supplement: Attachment — Submitted filename: Response to reviewers.pdf [file pcbi.1011487.s005.pdf]

Dear Editor,

We carefully considered the reviewers' suggestions to improve the manuscript's quality and clarity. We also performed additional simulations considering persistent inward current and different motoneuron size properties. Please find the detailed responses to the reviewers' comments below. Changes to the manuscript are highlighted in red font color. Also, note that the data repository is online now and contains the new simulation results. The data is currently accessible through a temporary link, and a permanent DOI is assigned as soon as the review protocol of the Uni Stuttgart's data management team is complete. If further changes are required, we will update the data repository accordingly.

temporary link (expires as soon as permanent link is activated):
https://darus.uni-stuttgart.de/privateurl.xhtml?token=27f7e10d-eda9-4811-90e8-b3a1aeb1125d

permanent link: https://doi.org/10.18419/darus-3686

Reviewer #1:

Summary:
This was a very interesting and enjoyable paper to read that proposed a novel mechanism of post-inhibition excitation from Ih to explain the excitatory rebound often seen following EMG/ motor unit firing rate suppression from an IPSP. The simulations were well done and the data well presented. I only have a few comments for consideration and several edits to the manuscript to make the text more precise and more relatable to a physiologist.

We want to thank the reviewer for her valuable feedback, which improved the quality of our manuscript considerably. We also appreciate your valuation of our study design.

Major comments.
1. There was no negative control performed in the simulation. That is, what would the PSF look like if an IPSP-EPSP from synaptic inputs was used in the model? Are there recordings of a hyperpolarized motoneuron that shows such a PSP profile? Perhaps the activation and inactivation characteristics of the Ih give a PSF profile that is closer to the human data than would a IPSP-EPSP profile from synaptic inputs? This could be elaborated on in the Discussion.

We can not exclude that in the experiment, the electrical stimulus of the nerve excited additional pathways (e.g. cutaneous receptors), causing an EPSP following the IPSP with some delay. Such a behavior can cause the observed rebound pattern. However, we did not model these pathways in this study to isolate the effect of intrinsic motoneuron properties. In our understanding, the model without h-current is the negative control. We have highlighted the possible reinforcing effects of excitatory pathways in the Discussion in 3.1 (new parts underlined):

"In the presented simulations, we did not consider other neuronal pathways that potentially contribute to the postinhibitory excitation. Importantly, the results shown in this work only consider the first poststimulus discharge. On this time scale, the involvement of a neural

pathway is unlikely. Nevertheless, we can not exclude that the electrical stimulation of the nerve excites additional pathways, which cause an excitatory postsynaptic potential with a short delay and reinforce the postinhibitory excitation. “

2. In the human data of Figure 1c, the amplitude of the post-inhibition excitation (peak of the PSF after 100 ms), is greater when the background firing rate is higher. This contrasts with the simulation results of Figure 4 where a lower drive produces a larger post-inhibition excitation. Can this discrepancy be explained?

We agree that the choice of exemplary PSF graphs should be unambiguous. In the original manuscript, we arbitrarily chose motor units from three trials to show that the strength of postinhibitory excitation is variable. We now use motor units from the same trial to avoid misleading conclusions from this figure, i.e., from one subject and 10% MVC.

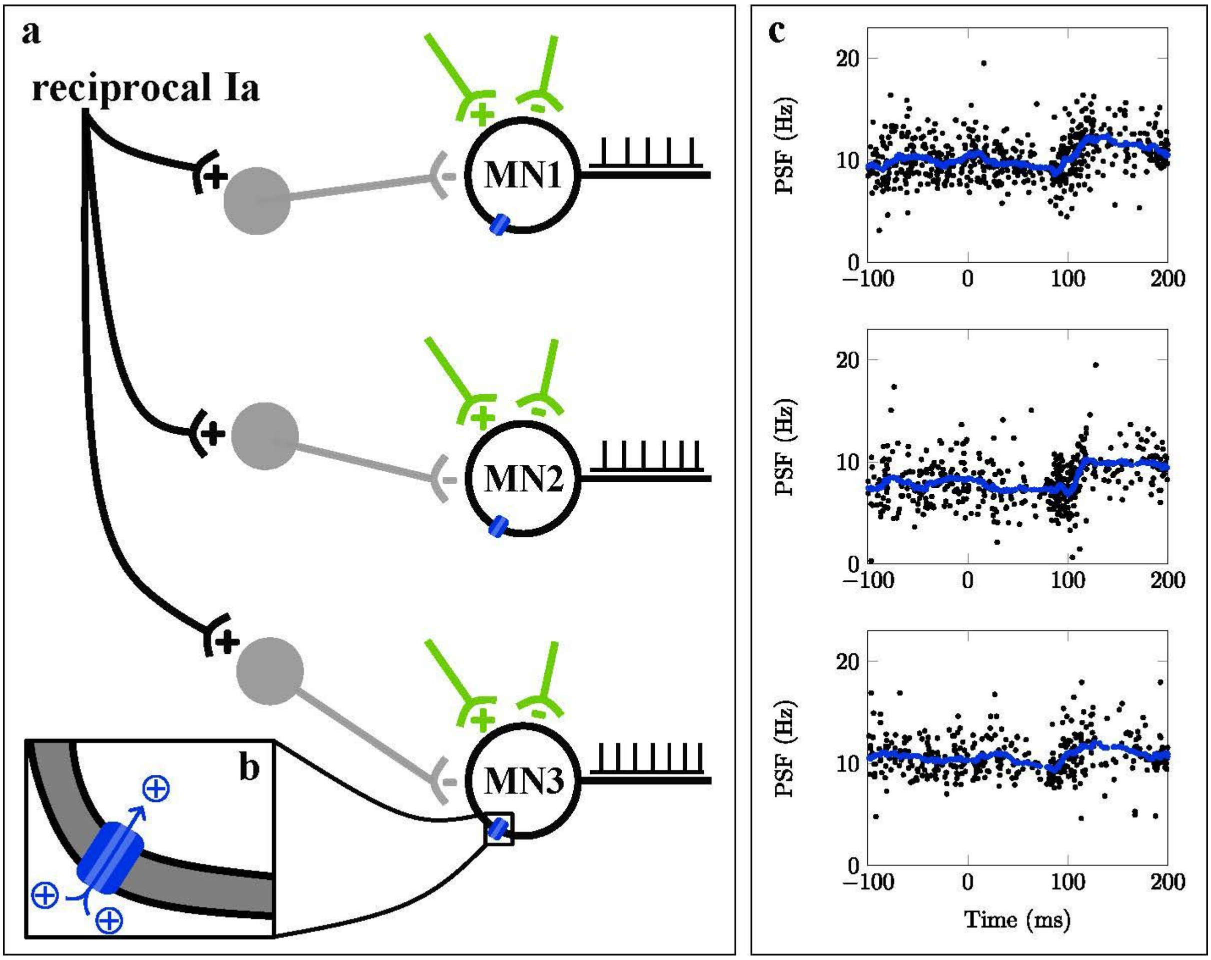

3. Details of the simulated IPSPs need to be added to the text and figures. It appears in Figure 2 in the human data, the IPSP lasts for 50-60 ms (duration of the decreasing PSF CUSUM below significance level). Likewise, in Figure 3 the duration of the silent period plus the duration of the decrease in the PSF CUSUM appears to be produced by an IPSP of 50 to 60 ms. It would be beneficial to plot the simulated IPSP over the PSF in Figure 3 to relate the firing behaviour of the motoneuron to simulated IPSP and give IPSP parameters used for

Figure 4. In Figure 5, the duration of the IPSP appears to be closer to 20 ms. Why was a 20 ms IPSP chosen and would similar results be obtained if an IPSP of 50-60 ms was used?

We always used the same IPSP duration (20 ms) in the simulations. The duration of the IPSP was determined empirically to match the duration of inhibition in the PSF-cusum with experimental data. Contrary to experimental PSF, the PSF of simulated motoneurons exhibited a silent period during the IPSP.

We added the IPSP profile to PSF in figure 3. Now the figure present the relation between the IPSP and discharge profile.

Thanks for your comment. We now explicitly explain the method to determine the IPSP duration and plotted the IPSP profile in Figure 3. In Figure 5, the drop in membrane potential closely follows the IPSP. In Figure 6, the same data is shown as in Figure 3, and the figure is already very busy, so we refrain from plotting the IPSP again.

Added in 4.4: "The inhibitory postsynaptic current was applied for 20 ms, as this, for the chosen amplitudes, achieved the minimum firing rate at a time comparable to the experiment."

Figure 3:

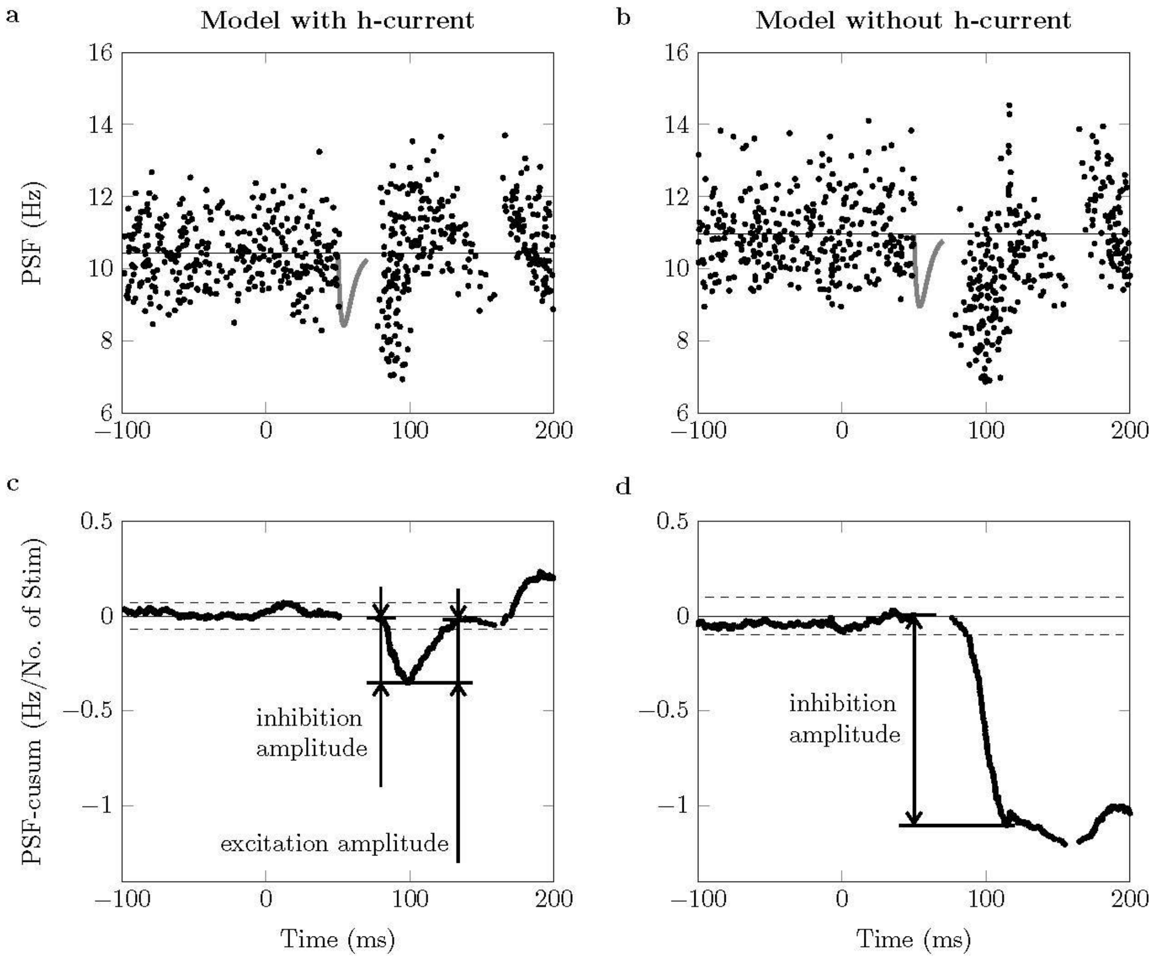

Additional comments are imbedded in the the uploaded PDF of the paper.

Thank you for pointing out how to make the manuscript more precise and understandable. We gladly considered your points and want to address some of them in detail:

- We added more details on the experimental procedure in the text, e.g. in Section 2.1, we added the underlined parts:
  "We examined the incidence and amplitude of postinhibitory excitation in 159 tibialis anterior motor units identified during the reciprocal inhibition experiment, i.e., electrical stimulation of the tibial nerve during sustained isometric contractions."

- You asked us if we could include more than 200 ms of poststimulus time and only 100 ms of prestimulus time in Figures 1, 2, and 3. We computed the PSF-cusum in a window with 200 ms pre- and 200 ms poststimulus time (300 ms for the simulation). A prestimulus time window that is at least the same length as the poststimulus time window is needed to estimate the significance of the peaks and troughs in the poststimulus time. We limit the poststimulus time to 200 ms since we focus on the phenomenon with a duration of not more than 150 ms. However, we agree that figures could focus more on the poststimulus time for better resolution. Therefore, we revised these figures to shorten the displayed prestimulus time to 100 ms and improve the visibility of the post-stimulus time window.

- In Figure 3, we added arrows for the amplitudes as in Figure 2 (see above).

- We refined the description of how inhibition and excitation amplitudes were determined (Section 4.5): "Troughs and peaks exceeding the error box are considered to represent significant inhibition and excitation responses, respectively. The response amplitude (inhibition or excitation) was defined as the difference between two turning points ~~the onset and the plateau of the peak or trough~~ in the PSF-cusum (Fig 2b, arrows). The turning points correspond to the start and end points of the monotonous slope of a significant reflex response and were determined manually. ~~The onset and the knee point of the plateau were determined manually.~~ We are aware that the duration of the inhibition is relevant. We did not consider the duration since we always used the same IPSC duration."

- We considered your suggestion to use Hz/No. of Stim as the unit of the PSF-cusum (instead of Hz/trigger). Note that we did not indicate all these changes in red color in the manuscript.

- Addressing your question on how the inhibition amplitude varied with mean drive and noise: In the figure below, we plotted the inhibition amplitudes determined from the PSF-cusum for different drive and noise levels. The emerging behavior is complex. As shown in Fig 6h, the total PSF-cusum results from the summation of the inhibitory and excitatory effects, which overlay in a certain area. This is why inhibition amplitudes are low whenever excitation amplitudes are high (cf. Figure 4 in the manuscript). This also explains why the inhibition first becomes stronger with higher IPSC amplitude (as expected) but then becomes smaller. Due to the h-channel activity, the "pure inhibition" can not be estimated from the PSF-cusum.

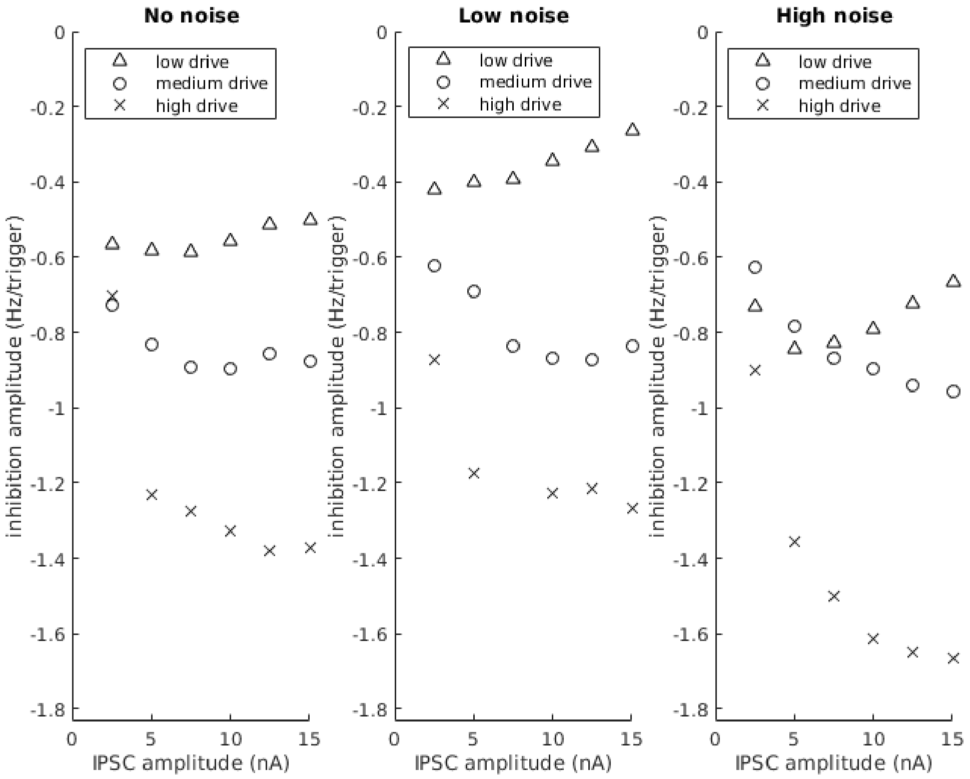

Reviewer #2: This is an interesting and well written study about motor neuron physiology and the role of intrinsic neuron's property on postinhibitory excitation following reciprocal inhibition. The paper combines human experiments and computer simulations to show some evidence on the involvement of H current on postinhibitory excitation. Despite the relevance of the topic, the opinion of this reviewer is that the current investigation neglects important biophysical aspects and thereby precludes a definite conclusion about the actual mechanisms behind postinhibitory excitation observed in human motor units.

We want to thank the reviewer for his/her valuable feedback, which we used to improve the quality of our manuscript. We agree that we can not make a definite conclusion about the origins of the postinhibitory excitation. With the help of your comments, we have emphasized this more in the text (see below).

- The authors discuss other potential mechanisms that would be involved in postinhibitory excitation, and they stated that they cannot rule out the involvement of some of them in behavior from human motor units. However, after reading the paper and the rationale provided by the authors one can have the idea that postinhibitory excitation in humans should be attributed to Ih. My opinion is that the authors don't have a strong evidence in favor of this argument and, therefore, they should revise the text to make clear that simulation results cannot ensure the real mechanism in human motor units involves Ih.

We thank you for your comment. We want to avoid creating the impression of proving the hypothesis that postinhibitory excitation is caused by hyperpolarization-activated channels.

However, hyperpolarization-activated channels are likely candidates contributing to postinhibitory excitation. Thus, we adapted the text in several places (new passages are underlined):

Discussion (3.1): “In the presented simulations, we did not consider other neuronal pathways that potentially contribute to the postinhibitory excitation. Importantly, the results shown in this work only consider the first poststimulus discharge. On this time scale, the involvement of a neural pathway is unlikely. Nevertheless, we can not exclude that the electrical stimulation of the nerve excites additional pathways, which cause an excitatory postsynaptic potential with a short delay and reinforce the postinhibitory excitation. “

Discussion (3.1): “It was shown that a considerable fraction of the experimentally recorded motor units showed characteristic clusters of both excitation and inhibition. The same patterns were observed for the simulated motoneurons with h-currents. ~~Hence, these finding provide evidence for h-current activity in human motor units.~~ Hence, these findings suggest h-current activity in human motor units. “

Last sentence of conclusion: “~~We conclude that h-currents serve as a modulatory system that is able to increase motoneuron excitability~~. According to the simulation results, it can be speculated that the h-current serves as a modulatory mechanism that increases the excitability of motoneurons.”

- In line with my previous comment, Bertrand and Cazalets showed evidence that postinhibitory excitation is mediated by Ca++-activated K+ current. The authors did not explore the role of Ca++-activated K+ current and other potential mechanisms (such as the deinactivation of K+ conductances). The Cisi-Kohn's model does not represent these molecular mechanisms, and therefore the simulation results are not comprehensive enough to be linked to a specific behavior observed experimentally.

Betrand & Cazalets found that replacing Ca++ in the medium affected only 3 out of 14 tested cells. In contrast, Cs+ had a considerable effect in all tested cells. This finding suggests that the rebound is most likely due to the h-current. Further, Ca++-activated K+ currents affect the AHP following a spike and would thus affect the interval after the one in which the IPSP occurs. However, this investigation focused on the interval during which the IPSP occurs.

The literature discusses numerous mechanisms contributing to the postinhibitory rebound. The h-current is repeatedly mentioned as the most probable mechanism and is therefore studied in this manuscript. Although we do not aim at a quantitative comparison due to the uncertainty of the model, the higher rebound amplitude in the experimental studies indicates that other mechanisms also play a role *in vivo*. However, the advantage of computer simulations is that individual mechanisms can be considered in isolation. Since the investigation of the h-channel requires a special new way of data analysis, we have decided not to include other mechanisms in this work.

- Manuel et al. (2007) showed that Ih causes the resonant behavior of the motoneuron membrane. However, they found that Ih (and resonant behavior) is typical of motoneurons innervating fast-contracting muscle fibers and that slow motoneurons do not exhibit resonant

behavior. Also, they found that the resonant behavior is primarily affected by Ca++ and Na+ persistent inward currents (PICs). My point is that the authors neglected the influence of PICs on this postinhibitory excitation. I am not confident that the lack of effect of PICs on reciprocal inhibition (as stated in the paper) is sufficient to adopt a model without PICs and their potential influence on the behavior the authors are trying to represent. Additionally, the authors described an S-type motoneuron, which was experimentally less susceptible to resonate and exhibit the voltage "sag" (overshoot).

Thank you for your comment. We performed additional simulations to address it. We added another supplementary figure to the manuscript showing the results (Fig S3).

We want to reply to your comment in two steps: addressing (i.) the PIC and (ii.) motoneuron size.

(i.) Manuel et al. 2007 found that slow PICs suppress the resonant behavior, and fast PICs enhance it. We added a slow and a fast PIC to the motoneuron model, using the dynamics employed by Manuel et al. in their study. We also used the same range of time constants (1ms and 100ms). However, we adapted the reversal potential for the fast PIC to the value of the sodium channel (120 mV). As in the study of Manuel et al., the slow PIC reduces the rebound. The fast PIC enhances the rebound but only when applied at the appropriate moment in the interspike interval. However, none of them completely suppresses the rebound. The results are shown in Fig S3b.

We also adapted the text accordingly.

Discussion (3.1): "Notably, other factors may modulate h-current mediated postinhibitory excitation. For example, persistent inward currents (PICs) can modulate h-current activity [24]. Adding a fast and a slow PIC to the motoneuron model, we could still observe stimulus-time dependent excitation (Fig S3b). However, Even though PICs were shown to play a minor role during Ia reciprocal inhibition [25], their role in postinhibitory excitation should be investigated in future studies. Further, due to their slow time dynamics, we expect no major changes in PIC activity during short inhibitory stimuli [26]. Importantly, modulations in h-current conductance have no influence on the characteristic opposing motoneuron response (Fig S 2b)."

(ii.) Manuel et al. 2007 reported that 39 out of 45 motoneurons showed resonant behavior, and the six motoneurons that did not were of slow type. They do not comment on the type of motoneurons included in the remaining 39. However, they started their discussion with the conclusion that "most" motoneurons show resonant behavior. We performed additional simulations with increased motoneuron size, corresponding to an FR-type motoneuron. In one simulation, we also increased the h-current conductance to account for potentially stronger resonant behavior in larger motoneurons. In the other simulation, we used the same conductance as in the default (S-type). Both motoneuron size and conductance only minimally affect the observed behavior.

We added a supplementary figure (Fig S3a).

We also adapted the text accordingly:

Limitations (3.2): “Although it is impossible to directly validate the utilized implementation, additional simulations showed that varying the h-channel parameters or motoneuron size does not affect the general behavior of the model (Fig S 2, Fig S3a).“

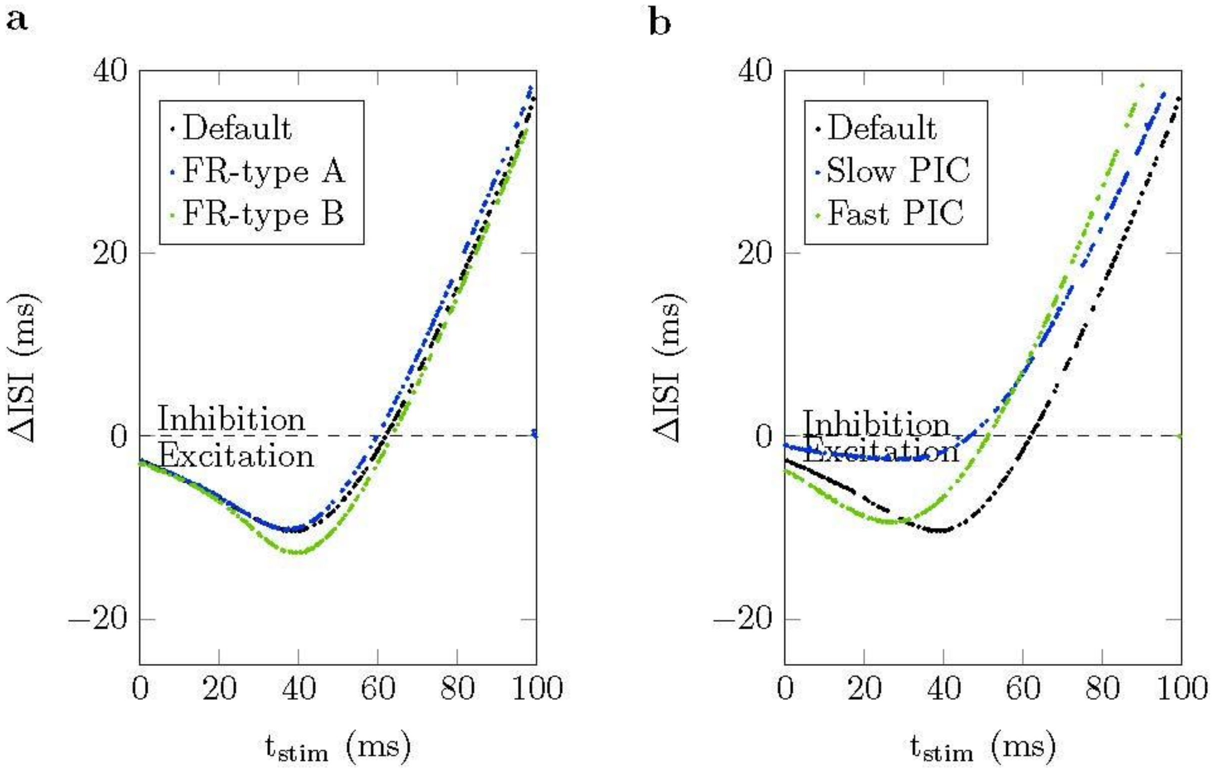

Fig S 3. Stimulus time-dependent duration of interspike intervals for different parameterizations of the motoneuron model. Change of interspike interval duration (Δ ISI) over time of stimulus (IPSP) application with respect to the last discharge (t_{stim}). Default parameters are shown in black. (a): Variation of the motoneuron size. FR-type motoneurons correspond to motoneurons with size-dependent parameters according to the mean values for FR-type neurons in Cisi and Kohn [17]. Version A and B employ a maximum h-channel conductance of $\bar{g}_{H,A} = 2$ mS/cm^2 and $\bar{g}_{H,B} = 4$ mS/cm^2, respectively. (b): Influence of persistent inward currents (PICs) injected into the dendrite compartment of the motoneuron model. PIC dynamics according to Manuel et al. [24]. Maximum PIC conductance 0.1 mS cm^{-2}. Slow PIC: time constant 100 ms, reversal potential 140 mV. Fast PIC: time constant 1 ms, reversal potential 120 mV. Baseline frequency 10 Hz, no noise, inhibitory postsynaptic current amplitude −10 nA.

- One weakness of the study is the lack of a proper sensitivity analysis regarding model's parameters. The authors varied some input parameters, such as excitation level, noise level, and inhibition amplitude. But it is not clear how changes in morphological properties or other electrophysiological properties would affect the postinhibitory excitation.

We performed simulations with a larger motoneuron (FR-type) and did not find qualitative changes in the behavior. For the details, please see the reply to the previous comment.

- After including the Ih current the f-I curve of the motor neuron changed considerably. The authors argued that the gain was maintained, but it is evident that the model with Ih is much more excitable than the model without Ih. The curve is upward shifted, so that to maintain the same mean discharge rate the neuron with Ih will require a much lower injected current. How the magnitude of the input excitation would affect Ih and the other ionic currents? Additionally, with the inclusion of Ih the electrophysiological properties of the motor neuron seem very different from a typical anesthetized S-type motoneuron (see Zengel's data). What would happen if you adjust the other ionic currents so that the model with Ih would resemble the f-I curve of a typical S-type motor neuron? Also, other electrophysiological properties are important to validate the model, such as the AHP amplitude and duration, rheobase, etc.

Including an additional ion channel will always change the behavior of the motoneuron model. In the case of the h-current, the excitability of the motoneuron increases. We decided against adapting the other channels, as we would no longer be able to attribute the changes in behavior directly to the h-current. Instead, we reduced the injected current to compensate for the higher excitability. This yielded very similar time courses of the channel gates when comparing interspike intervals of identical length. To better visualize this, we replaced supplementary figure 1 with the figure inserted below. Calculating the Euclidean norm of the difference of the gating variables shows that, on average, they deviate by less than 10%. Thus, we consider the two models to behave sufficiently similar.

Limitations (3.2): "To compare the model with h-currents and the knock-out model the input current was adjusted, i.e., both models operate at comparable points of their current-frequency relation. This guarantees that differences are exclusively attributed to h-currents. Specifically, the membrane potential and the ion channel gating variables deviate from each other by less than 10 % on average for identically long interspike intervals (Fig S 1)."

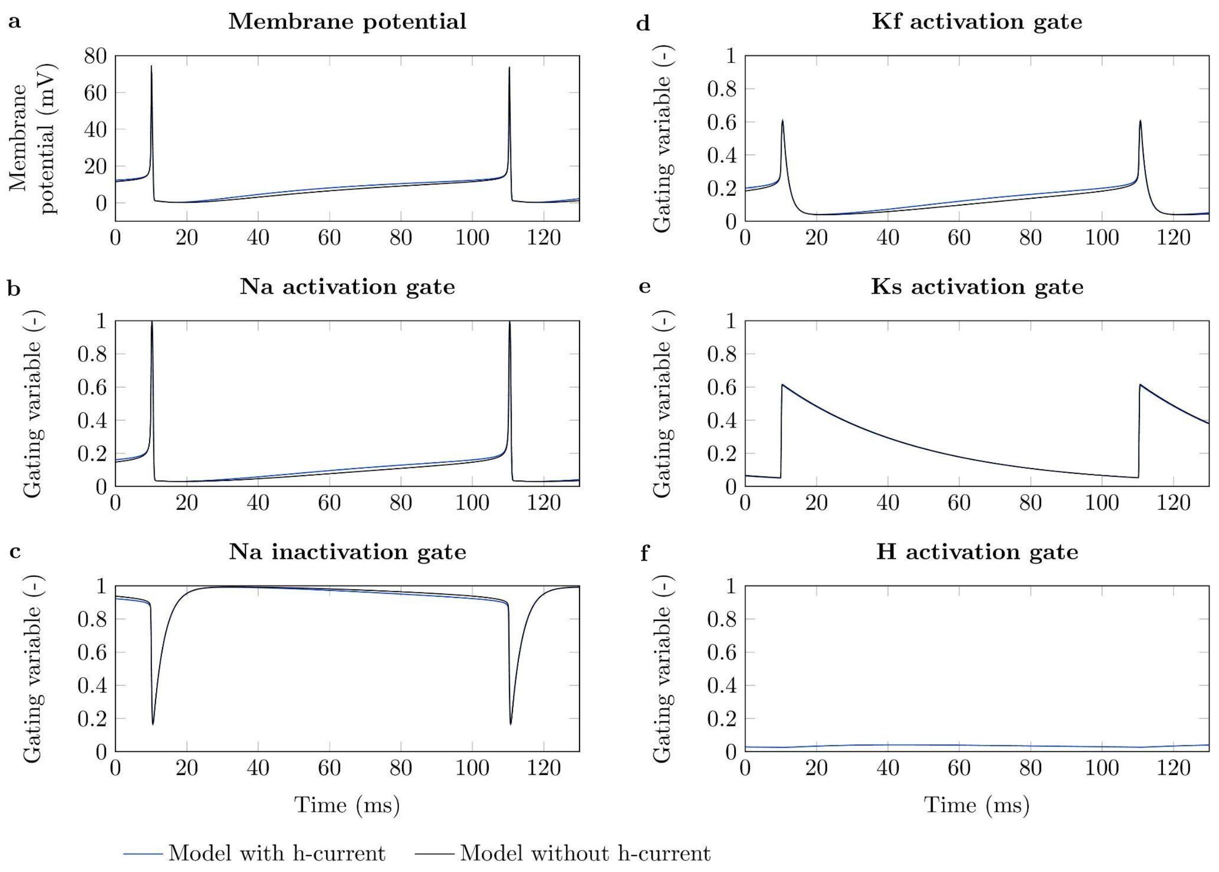

Fig S 1. Motoneuron model gating variables. Motoneuron model gating variables for one interspike interval (duration 90.3 ms) of the model with (blue) and without (black) h-current. Shown are membrane potential (a), sodium channel (Na) activation gate (b), Na inactivation gate (c), fast potassium channels (Kf) activation gate (d), slow potassium channel (Ks) activation gate (e) and h-channel (H) activation gate (f). The gating variables are defined as described in Negro and Farina [18] and Röhrle et al. [39].

- The condition where postinhibitory excitation was frequently observed in simulations seems quite stringent (and probably non-physiological). Absence or low noise is unlikely in the motor system. What happens if the neuron is discharging closer to its threshold? Do you think the role of noise is different in the latter condition? Again, this comment is in line with my previous argument that the simulations are not comprehensive enough to guarantee that the mechanism behind postinhibitory excitation was fully explored.

We agree that the effect of noise is different when the motoneuron operates close to its threshold. Thus, we combined three drive and three noise levels in the simulations. The neuron operates close to its threshold in the “low drive” condition. As shown in Figure 4, noise reduces the excitability. This has consequences for the potential functional role of h-currents. Thus, we adapted the manuscript in Section 3.3 (Functional significance and future directions):

“We showed that h-current mediated postinhibitory excitation is most pronounced when a motoneuron operates close to its recruitment threshold and for strong inhibitory stimuli. This is interesting regarding the functional significance of h-currents. ~~Thus,~~ We speculate that

h-currents temporally increase the excitability of a motoneuron and potentially protect a motor unit from derecruitment."

- The magnitude of postinhibitory excitation obtained from computer simulations is very different from those obtained in the experiments. The authors should discuss the potential causes for these quantitative differences. Do you think other mechanisms would be involved? Can you guarantee that the set of parameters are optimally chosen?

This point summarizes many of the above comments. Even though our chosen set of parameters is compatible with what we know from physiology, there remains uncertainty. This is also why we do not make quantitative statements on how much the h-currents contribute to postinhibitory excitation. However, it is noticeable that the amplitude is higher in the experimental studies. We discuss several causes for this discrepancy in Sections 3.1 and 3.2: PICs, (calcium-activated) potassium conductances, low threshold sodium conductances, delayed recovery of the sodium inactivation gate (anode break excitation), T-type calcium channels, NMDA receptors and neuronal pathways (which would have to be identified).

To make our point clearer, we added a paragraph to the manuscript in the Discussion (3.1):

"Due to the uncertainty of the parameters, we refrain from a quantitative comparison with the experimental data. Nevertheless, the notably higher excitation amplitudes in the experimentally recorded motor units indicate that h-currents are not the only mechanism playing a role in the postinhibitory excitation phenomenon. "

Reviewer #3: This study aims to better understand the processes behind a rebound of motoneuron firing rate after receiving inhibitory synaptic inputs. This rebound has been observed in vivo in humans on motoneuron firing rates extracted during isometric contractions by combining high-density electromyography recordings and decomposition algorithms based on blind-source separation.

As it is not possible to directly record in vivo in humans the synaptic inputs and inward currents causing these changes in firing rate, the authors used a biophysical model of motor neurons to directly test their hypotheses and reproduce the motoneuron firing activity observed in experiments. Their hypothesis is that 'h-currents' transiently change the excitability of the motor neuron, which in turn increase the probability of firings. The study appears well-conducted, with the use of classic biophysical models developed and refined in parallel by several teams (Heckman Powers Binder…, Cisi Kohn Elias…, Negro Farina Dideriksen…) and the conclusions are convincing. I have therefore only minor questions and comments.

We thank the reviewer for his feedback and valuation of our study design. Your suggestions were very interesting. Please also find our answers below.

Minor comments:

1.
Motoneuron firing rates have been extracted while the participants were performing a force-matched task with visual feedback. I was wondering whether the authors observed a short drop in force with the nerve stimulation followed by a sharp increase of force to correct the error in tracking and go back to the target? If yes, could the observed rebound in firing rate be caused by a transient change in net synaptic drive proportional to the force, with a pattern of firing rate matching the force profile?

In general, adding variations in force on the plots and the results would be appreciated to convince the reader that your rebound is probably due to other factors, such as inward currents.

We elicited reciprocal inhibition on antagonist muscle using low-intensity electrical stimulations. To this end, we identified the stimulation intensity to induce an H-reflex with a peak-to-peak amplitude of 10% of the maximum M-wave of the homonymous muscle. This is where the stimulation intensity was on the ascending part of the H-reflex recruitment curve, and the M-wave was absent or of negligible amplitude. With this stimulation intensity, we avoid inducing a sharp drop in force and, thus, a silent period in motor unit firings. Yet, we cannot claim that the stimulation is fully selective and excites homonymous Ia only. However, we can ensure that the stimulation did not elicit strong contraction force but subtle reflex movements. Please find an example of one force recording below.

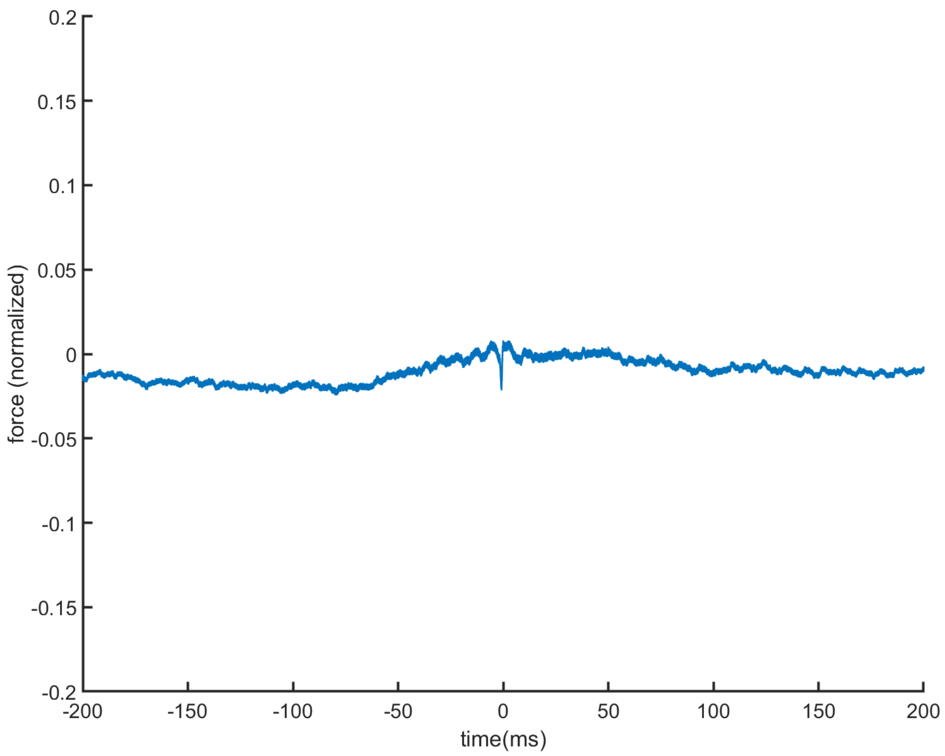

2.
The duration of the contractions during the experiment is quite long (250s), at non-negligeable level of force (10 to 20%MVC). Beside fatigue, it is standard to observe a slow decrease in firing rate during long plateaus of force due to accommodation or adaptation phenomenon (Revill & Fuglevand, 2011, J Neurophysiol). Such variations are link to slow changes in the intrinsic properties of motor neurons and the inactivation of ionic channels. I was therefore wondering whether you observed a time effect on the rebound after the stimuli, with different responses at the beginning and the end of the contraction? Such changes could be linked to other factors than those tested by the authors.

It is a good point to see the effect of fatigue on reflex and rebound gain. However, we cannot examine this with the available data. This is because the duration of recording was not long enough to split the data into multiple segments each of which with a minimum of 100 stimuli. The minimum number of stimuli for a reliable estimation of reflex amplitude should be at least ~100 stimuli. Indeed, in some subjects, it might be possible to divide the recording (when we were able to deliver a large amount of stimuli). However, such an analysis is beyond the scope of this manuscript.

3.
All the simulations are performed on a single motor neuron with fixed properties such as the rheobase, soma/dendrites radius, length, and resistance. I understand this choice for the sake of simplicity, but it would be interesting to have a sense on the impact of the size of the motor neuron on the presence of a rebound or not after an inhibitory stimulus. Moreover, having an idea on the effect of this phenomenon at the population level would help to infer potential functional consequences.

We agree that investigating postinhibitory excitation on the motoneuron population level would be very interesting. A similar point was also raised by reviewer #2. We performed additional simulations with a larger motoneuron size and with PICs added to the motoneuron model. Both affected the amount of the observable postinhibitory excitation but not the qualitative behavior. We show these results in the new supplementary figure 3.

4.
It is sometime difficult to understand the analyses and the results without going to the method at the end of the manuscript. This is annoying when the results come first in the paper. I would recommend adding more details about the methods in the result section to improve the flow of the story, and simplify the life of the reader.

This point was also raised by Reviewer #1, who suggested adding details on the methods at several points within the Results section. We followed her suggestions and hope that the manuscript is more readable now.

Specific comments:
Results: L. 109 the ‘e’ is missing in Therefore
