# Supplementary material for: Postinhibitory excitation in motoneurons can be facilitated by hyperpolarization-activated inward currents: A simulation study
Source: PLoS Comput Biol. 2024 Jan 19;20(1):e1011487. doi: 10.1371/journal.pcbi.1011487 (PMC10843122; doi:10.1371/journal.pcbi.1011487)

Additional comments.
Thank you for the additional clarification on IPSP duration. The reason why I assumed the IPSP's in Figure 3 were around 50 to 60 ms was that in the data below from Norton et al., 2008 (left graphs), a post-synaptic potential that contains a ~60 ms IPSP that is followed by an EPSP or rebound excitation (A: top left, rat data) produces a clustering of action potentials at lower frequencies on the repolarization phase of the IPSP (at black arrow) that is also seen in the human firing rate profiles (C: top right). This clustering is also seen in your human data of Figure 2 near or just before 100 ms. Action potentials on the repolarization phase are also seen in the simulated IPSP only data from Figure 8 in Turker and Powers 1999 (right graphs, 50 ms IPSP). Note, most post-synaptic potentials recorded in animals without anesthetic are > 20 ms given that many interneurons possess persistent inward currents, which can produce prolonged firing (and long motoneuron IPSPs) in response to a single afferent stimulation (e.g., Cavarsan et al. J Physiol 2023).

It's not clear why, in your model in Figure 3, action potentials are not being triggered on the repolarization phase of the IPSP (at blue arrows), where the rapid increase in membrane potential should trigger a spike. As shown in B from Norton et al., 2008, the repolarization phase of the simulated IPSP produces a rapid increase in membrane potential that triggers a spike (at blue arrow), especially after the spike has been delayed by the IPSP. Thus, in Figure 3 it is not clear why your model doesn't produce action potentials during the repolarization phase of the IPSP or even when the membrane potential returns to baseline?

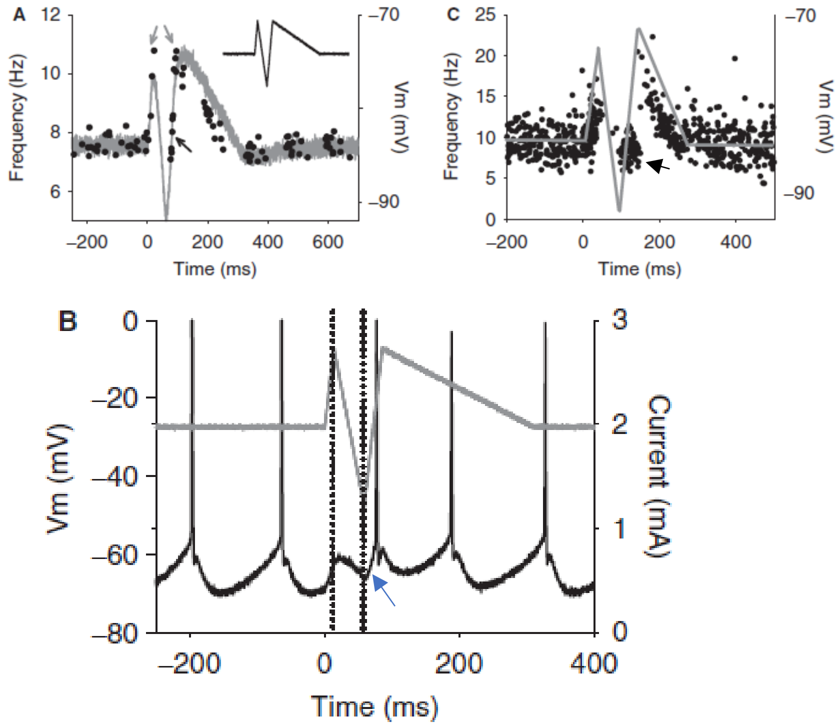

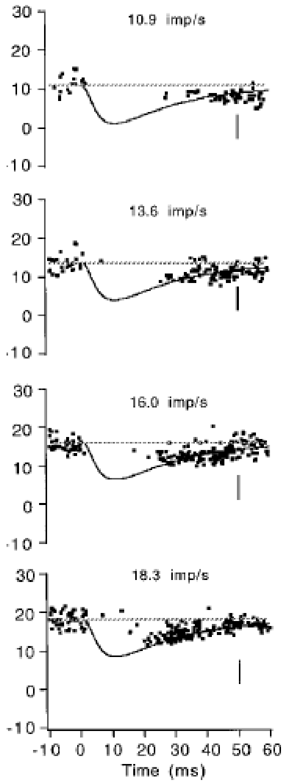

Turker and Powers 1999

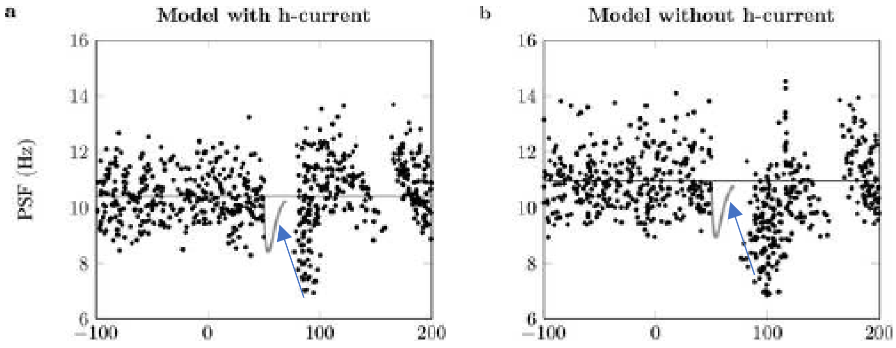

One explanation for the lack of action potentials during IPSP repolarization may come from the data shown in Figure 5. In the green trace, after the IPSP is finished, the membrane potential trajectory should rapidly increase after the AHP is over (see red line) because of the tonic depolarizing drive. This Vm acceleration should trigger a spike as in the no IPSP (black) condition. However, in the green Vm trajectory after the IPSP, it appears that the AHP duration (the continued slow rise in membrane potential, circled area) is being extended by ~25 ms, resulting in a prolonged silent period. It is not clear why the AHP after the 20 ms IPSP (and thus interspike interval) is being extended in this way? The only instance where the interspike interval should be lengthened by the IPSP is when the start of the IPSP occurs just before the expected spike so that spiking is stopped during the hyperpolarization phase of the IPSP (see Norton figure above) or during the initial repolarization phase (see Turker and Powers figure above). It is also not clear why the AHP duration is also extended (but to a smaller amount) in the blue trace after the IPSP is over. In this case, the motoneuron should fire at the same time as in the no IPSP (black) condition. Is the AHP (or IPSP) being extended by some unexplained factor?

Given that the membrane potential after the IPSP and AHP should accelerate faster because of the steady depolarizing drive producing the tonic firing, a longer IPSP duration should be used to replicate the human PSF data. An IPSP with an onset latency of ~50 ms and duration of ~60 ms would produce a silent period (or lowered firing rates) between 50 and 70 ms during the hyperpolarizing and initial repolarizing phase of the IPSP, and a large clustering of action potentials between 70 and 110 ms during the later repolarization phase of the IPSP. Moreover, the clustering of action potentials during the later repolarization phase of the IPSP may be facilitated by a velocity-dependent lowering of the fast sodium spike threshold (Kuo et al., J Physiol 2006). For instance, the rate of rise of the membrane potential during the repolarization phase of the IPSP is faster than the rate of rise at the end of the AHP just before the membrane potential reaches spike threshold. Thus, it would be easier to evoke spikes during the fast-rising repolarization phase of the IPSP to produce clustering of action potentials (as seen in your Figure 2).

Although the duration of the IPSP and AHP trajectory should potentially be changed in the model, in the end this should not affect the Ih results where the added depolarization from Ih should help amplify the velocity dependent lowering of the fast sodium spike threshold during the repolarizing phase of the IPSP to produce a greater rebound excitation.

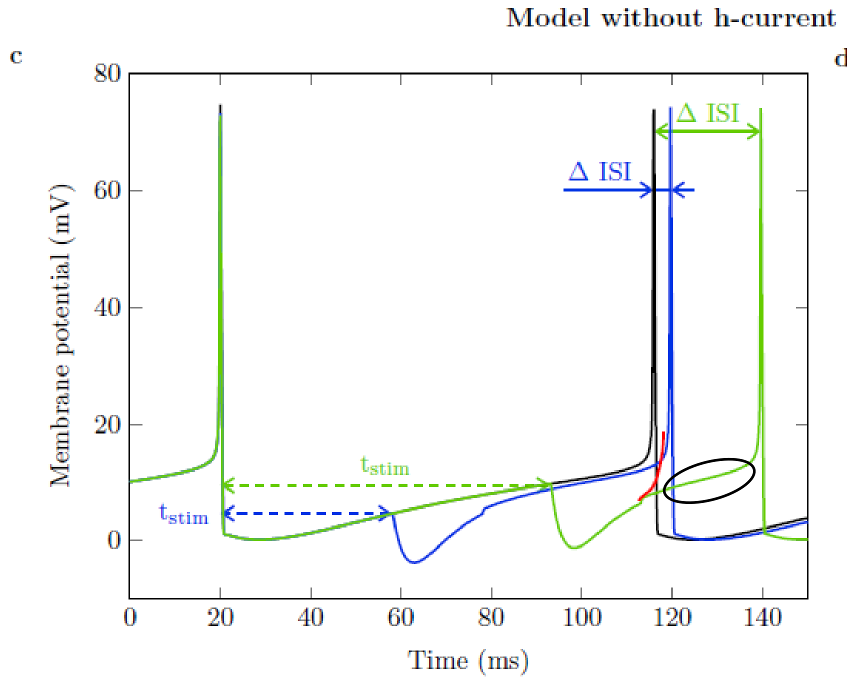

Supplement: Attachment — Submitted filename: PLoS review D-23-01428_R1.pdf [file pcbi.1011487.s006.pdf]
