# Supplementary material for: Postinhibitory excitation in motoneurons can be facilitated by hyperpolarization-activated inward currents: A simulation study
Source: PLoS Comput Biol. 2024 Jan 19;20(1):e1011487. doi: 10.1371/journal.pcbi.1011487 (PMC10843122; doi:10.1371/journal.pcbi.1011487)
Supplement: Attachment — Submitted filename: response_to_reviewers.pdf [file pcbi.1011487.s007.pdf]

Dear Prof. Gorassini,

Thank you for your helpful comment. We realized that we were unclear in communicating the difference between the inhibitory postsynaptic current (IPSC) and the inhibitory postsynaptic potential (IPSP). We apologize for that and made changes to the manuscript to avoid confusion for the readers.

In the model, we injected an inhibitory postsynaptic current (IPSC) to induce an IPSP. Thereby, the duration of this current kernel was fixed to 20 ms. The IPSP caused by this current has a duration of ~50 ms, due to the integrative behavior of the motoneuron membrane. The exact duration of the IPSP depends on the timing of the current injection. The change in membrane potential in response to a particular current injection depends on the momentary value of the membrane potential and active conductances. This can be seen in Figure 5. The black trace corresponds to an undisturbed reference interspike interval, i.e., only a constant depolarizing current is applied. The inhibitory current (IPSC) was applied for 20 ms in the blue and the green trace. Even after the release of the current, the membrane potential does not directly "jump" back but gradually increases until it matches the black trace. We illustrated this behavior in the figure below. In red we marked the duration of the injected current (IPSC); in yellow we marked the duration of the IPSP.

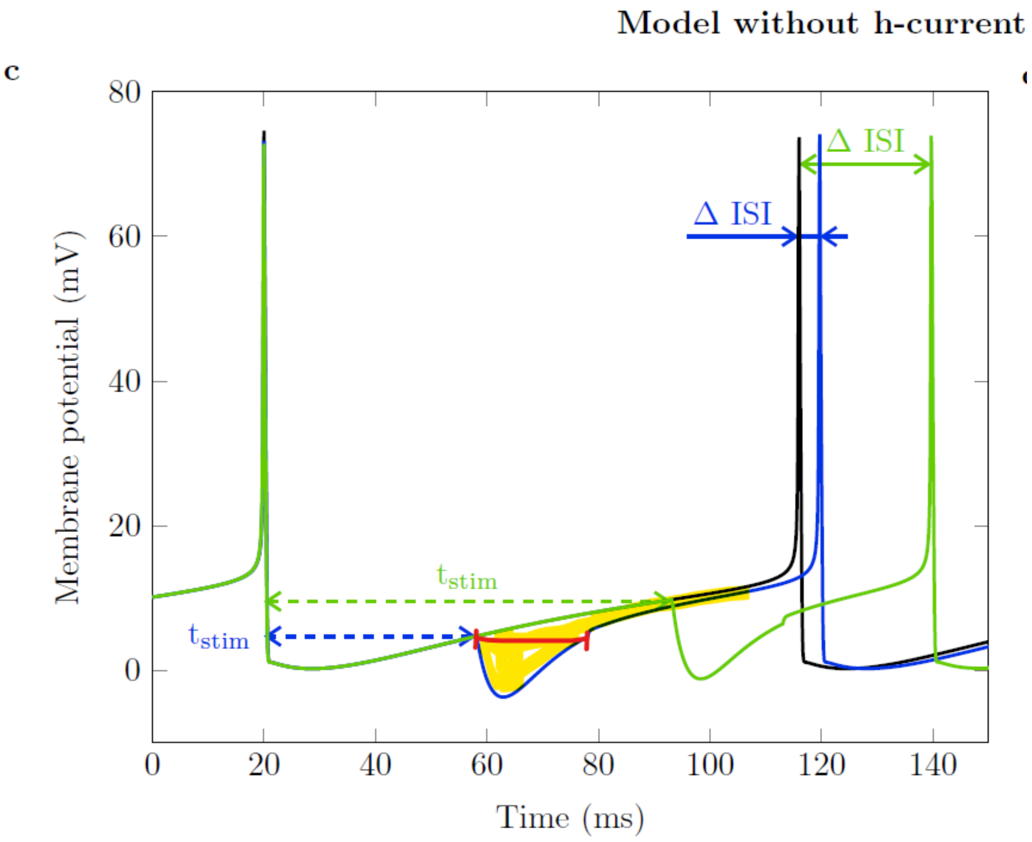

The time course for the rising phase of the AHP you drew in red in the Figure, is impossible with the chosen inputs. The black trace corresponds to the model response for a purely excitatory (constant) input. Therefore, the increase in membrane potential will be similar to the black trace when the IPSP is over.

In Figure 3, we only showed the IPSC, because it is the input for the simulations and is constant for each simulation. However, we agree that it is common to plot the IPSP, especially in experimental (*in-vitro*) studies. Hence, Figure 3 might cause confusion to some readers. Therefore, we added a schematic IPSP profile to the figure. Now, the figure shows both the relation between injected current and resulting IPSP and the relation between IPSP and the firing behavior.

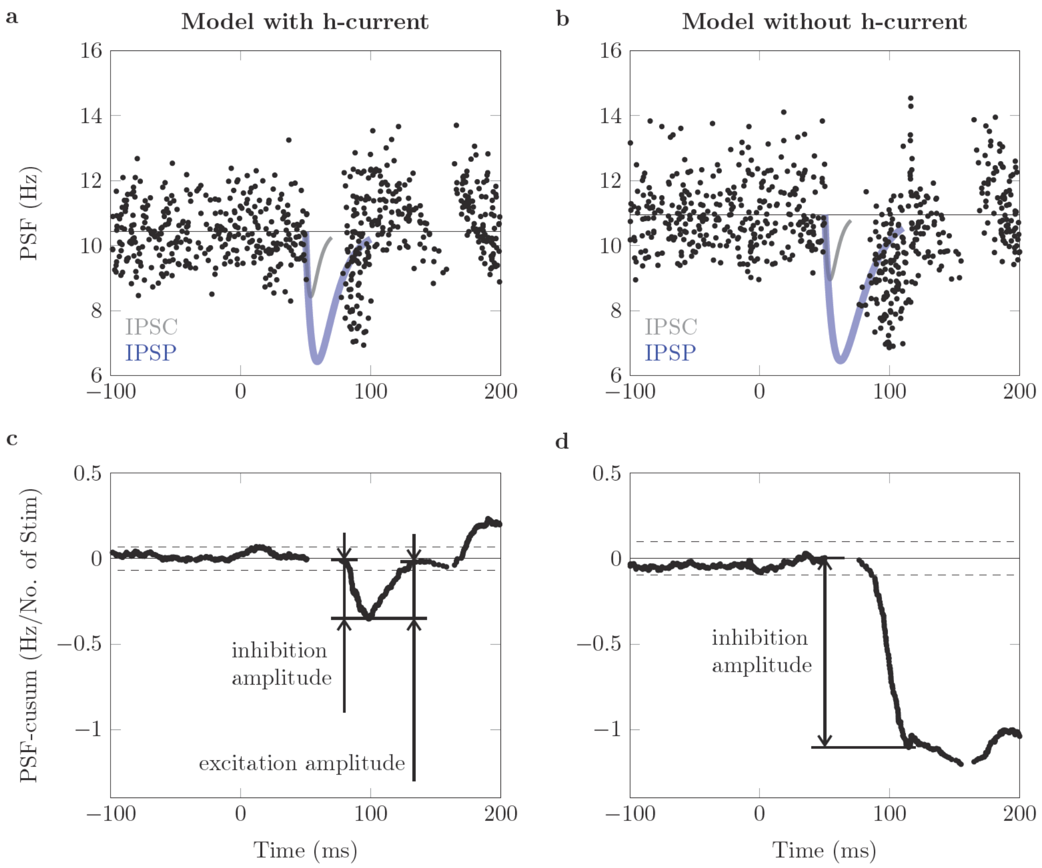

Fig 3. Peristimulus analysis for simulated motoneurons. (a): Peristimulus frequencygram (PSF) for a simulated neuron with h-current. (b): PSF for a simulated neuron without h-current. <u>In (a) and (b), the injected inhibitory synaptic current (IPSC) and the schematic trajectory of the induced inhibitory postsynaptic potential (IPSP) are depicted in gray and blue color, respectively. The actual time course of the membrane potential depends on the membrane potential value and the size of other inputs at IPSC application time.</u> (c): PSF cumulative summation (PSF-cusum) for a simulated neuron with h-current. (d): PSF-cusum for a simulated neuron without h-current. Solid horizontal lines show prestimulus mean values and dashed lines mark the significance threshold for reflex responses. Arrows show the distance between two manually determined turning points in PSF-cusum, i.e., inhibition and excitation amplitude.

In addition, we clarified the descriptions in the manuscript (new text is underlined):

3.2 Discussion/Limitations:

Reciprocal inhibition indirectly stimulates the motoneurons through the afferent nerve. The magnitude of the ~~IPSP in the motoneuron~~ inhibitory input to the motoneuron caused by interneurons is unknown. To compare the simulations and experimental data, the amplitude of the injected current was chosen such that the inhibition amplitudes and latencies determined from the PSF-cusum are in the same range as for the experimental study. In this way, we assume that the injected current produces an IPSP comparable to the *in-vivo* conditions. Nevertheless, a quantitative comparison of the postinhibitory excitation amplitudes of *in-silico* and *in-vivo* motoneurons is beyond the scope of this study.

4.4 Methods/Simulation of reciprocal inhibition:

The inhibitory postsynaptic current was applied for 20 ms, as this, for the chosen amplitudes, achieved the minimum firing rate at a time comparable to the experiment. The current-induced IPSP usually last longer than 20 ms (Fig 3a, b).
